# Molecular Profiling of Polish Pediatric Patients with Epilepsy: A Single-Center Diagnostic Experience Using Next-Generation Sequencing

**DOI:** 10.3390/genes17020133

**Published:** 2026-01-27

**Authors:** Beata Chałupczyńska, Elżbieta Ciara, Paulina Halat-Wolska, Agnieszka Pollak, Piotr Stawiński, Dorota Jurkiewicz, Dorota Piekutowska-Abramczuk, Marzena Gawlik, Justyna Pietrasik, Agata Cieślikowska, Dorota Wicher, Agata Ulatowska, Dominika Jedlińska, Julita Borkowska, Dariusz Chmielewski, Dorota Dunin-Wąsowicz, Katarzyna Kotulska-Jóźwiak, Krystyna Chrzanowska, Agnieszka Madej-Pilarczyk

**Affiliations:** 1 Department of Medical Genetics, The Children’s Memorial Health Institute, Member of the European Reference Network ITHACA, 04-730 Warsaw, Poland; b.chalupczynska@ipczd.pl (B.C.); p.halat@ipczd.pl (P.H.-W.); p.stawinski@ipczd.pl (P.S.); d.jurkiewicz@ipczd.pl (D.J.); d.abramczuk@ipczd.pl (D.P.-A.); m.gawlik@ipczd.pl (M.G.); j.pietrasik@ipczd.pl (J.P.); a.cieslikowska@ipczd.pl (A.C.); d.wicher@ipczd.pl (D.W.); k.chrzanowska@ipczd.pl (K.C.); a.madej-pilarczyk@ipczd.pl (A.M.-P.); 2 Department of Medical Genetics, Warsaw Medical University, 02-106 Warsaw, Poland; agnieszka.pollak@wum.edu.pl; 3 Department of Neurology and Epileptology, The Children’s Memorial Health Institute, Member of the European Reference Network EpiCARE, 04-730 Warsaw, Poland; a.ulatowska@ipczd.pl (A.U.); d.jedlinska@ipczd.pl (D.J.); j.borkowska@ipczd.pl (J.B.); d.chmielewski@ipczd.pl (D.C.); d.dunin-wasowicz@ipczd.pl (D.D.-W.); k.kotulska@ipczd.pl (K.K.-J.)

**Keywords:** rare disease, epilepsy, Dravet syndrome, developmental and epileptic encephalopathy, next-generation sequencing, genetic testing, molecular profile, pediatric population

## Abstract

**Introduction:** Epilepsy syndromes show marked clinical and genetic heterogeneity, with numerous functionally diverse genes involved in their etiology. Next-generation sequencing (NGS) has facilitated the identification of many monogenic epilepsy syndromes and enables earlier, more accurate diagnosis in pediatric patients. **Materials and Methods:** This study analyzes the molecular profiles of 87 pediatric patients with various forms of epilepsy in whom pathogenic or likely pathogenic variants were identified. Next-generation sequencing (NGS) using multi-gene epilepsy panels or whole-exome sequencing (WES) was performed. **Results:** A total of 88 pathogenic or likely pathogenic variants were detected in 48 epilepsy-related genes; 30 variants occurred de novo. *SCN1A* and *KCNQ2* were the most frequent contributors (12.6% and 9.2%, respectively). The highest percentage of positive diagnoses (48%) was observed in patients with developmental and epileptic encephalopathy (DEE), with variants identified in genes including *ALG13*, *ATP1A2*, *CACNA1A*, *CDKL5*, *CHD2*, *GABRG2*, *ITPA*, *KCNQ2*, *PCDH19*, *SCN1A*, *SCN2A*, *SCN3A*, *SCN8A*, *SMC1A*, *SPTAN1*, *STXBP1*, and *UBA5*. Pathogenic variants in *ANKRD11* were found in four patients with KBG syndrome, while other genes appeared sporadically. **Conclusions:** Targeted massively parallel sequencing is an effective diagnostic tool for pediatric epilepsy. The presence of numerous single-case findings highlights the high genetic heterogeneity of epilepsy. This approach enabled more precise diagnoses that would not have been achieved through clinical evaluation alone, underscoring the importance of genetic testing for prognosis and treatment planning in pediatric patients with unexplained epilepsy.

## 1. Introduction

Epilepsy is a common chronic disorder of the central nervous system and one of the most frequent complex neurological conditions in childhood. Its incidence shows a distinctly age-dependent pattern, with the highest rates observed during the first year of life, underscoring the importance of pediatric epilepsy as a critical area of focus in clinical care. Epilepsy arises from heterogeneous etiologies, including genetic: structural, metabolic, immune and non-genetic external/secondary epilepsies: infectious, stroke, traumatic brain injury and unknown causes. In nearly half of all cases no definitive etiology can be identified. Nevertheless, a substantial proportion of childhood-onset epilepsy have a genetic basis [1,2,3], and it is estimated that up to 40% of patients with severe epilepsy have a single-gene etiology [4].

The genetic background of epilepsy is highly complex, encompassing both monogenic and polygenic forms. Single-gene variants are widely recognized as a predominant pathogenic mechanism underlying many genetic epilepsies. With expanding knowledge of seizure pathophysiology, the number of genes implicated in monogenic epilepsy continues to grow rapidly. Currently, according to the OMIM database, 111 genes are associated with the DEE phenotype. These DEE-associated genes are listed in Supplementary Table S1. At the same time, molecular genetic testing has revealed not only extensive genetic heterogeneity within specific epilepsy syndromes but also marked phenotypic pleiotropy, whereby alterations in the same gene can lead to a broad range of epileptic and neurodevelopmental manifestations [5]. Childhood monogenic epilepsies associated with developmental delay therefore represent a wide spectrum of disorders involving diverse biological pathways—from ion channels and receptors to genes regulating cellular metabolism and signaling.

Advances in high-throughput sequencing technologies, particularly next-generation sequencing (NGS), have substantially expanded our ability to detect pathogenic variants and have become a cornerstone of clinical diagnostics of rare diseases. NGS-based approaches, such as targeted multigene panels and exome sequencing, enable the rapid identification of disease-causing variants, especially in patients with early-onset, treatment-resistant seizures and additional comorbidities. However, large-scale sequencing also generates extensive datasets that often include variants of uncertain significance, complicating clinical interpretation and underscoring the need for further refinement of molecular diagnostic strategies.

Integrating clinical and genetic data is crucial for understanding disease mechanisms, improving diagnostic accuracy, identifying novel pathogenic genes or biomarkers, and expanding the known genetic spectrum of epilepsy. Such integration also facilitates prediction of disease trajectory and prognosis through longitudinal follow-up and supports the development of precision medicine strategies tailored to specific genetic etiologies. Establishing an accurate molecular diagnosis as early as possible is essential for prognosis and appropriate counseling, and in monogenic epilepsies may also guide the selection of individualized therapeutic interventions targeting the underlying disease mechanism. Because the nature of the molecular defect directly influences both disease severity and therapeutic options, identifying the exact pathogenic variant, rather than only the affected gene, is critical for effective clinical management [6].

In this context, the present study evaluated 87 pediatric patients with epilepsy using NGS-based diagnostic methods, with the aim of identifying pathogenic or likely pathogenic variants, characterizing their clinical features, and assessing the effectiveness as well as the current limitations of molecular diagnostic strategies in pediatric epilepsy.

## 2. Materials and Methods

### 2.1. Patients

The study group consisted of 87 Polish patients with unexplained pediatric-onset epilepsy, referred to the Department of Medical Genetics of the Children’s Memorial Health Institute (CMHI) between April 2015 and May 2025. Structured clinical information was collected using a standardized form completed by the referring clinician or extracted from the medical records. Only patients under 18 years of age were eligible for inclusion. To classify each patient’s epilepsy, the following categories were considered: age at seizure onset, seizure type, suspected epileptic encephalopathy, suspected Dravet syndrome, refractory seizures, suspected channelopathy/receptoropathy, suspected metabolic epilepsy, structural brain anomalies and intellectual disability (ID)/developmental delay (DD). All participants or their legal guardians provided written informed consent to genetic analysis and participation in the study. The project was approved by the institutional bioethics committee.

### 2.2. Genetic Testing and Data Analysis

Genetic testing in all 87 individuals was performed using genomic DNA automatically extracted from peripheral blood leukocytes with either a MagNA Pure LC 2.0 (Roche Diagnostics, Risch-Rotkreuz, Switzerland) or a MagCore Nucleic Acid Extractor HF16Plus (RBC Bioscience, New Taipei City, Taiwan), according to the manufacturer’s protocol.

#### 2.2.1. Next-Generation Sequencing and Variant Interpretation

About 50 ng of high-quality genomic DNA was used for next-generation sequencing (NGS). NGS was performed using either the original CMHI sequencing panel (CMHI1000) of over 1000 clinically relevant genes (Twist Bioscience, San Francisco, CA, USA) (Supplementary Table S2), TruSight One (TSO) sequencing panel (Illumina, San Diego, CA, USA), or whole exome sequencing (WES)*,* constructed with the Twist Human Core Exome + Human RefSeq Panel kit (Twist Bioscience, San Francisco, CA, USA) to covering 99% of the protein-coding genes. Libraries were sequenced on the NovaSeq 6000 system (Illumina, San Diego, CA, USA) according to the manufacturer’s protocol. The average read depth was 129, with over 95% of the target regions covered at a minimum depth of 20-fold. The ‘Fold 80 base penalty’ parameter was used to assess coverage uniformity, and its average value is 1.28.

The most recent version of the epilepsy gene panel included 776 genes associated with developmental and epileptic encephalopathy and other epileptic syndromes (Supplementary Table S1) and was used as the primary test. Over the 10-year recruitment period, the panel was regularly updated to incorporate newly identified epilepsy-associated genes based on current information from Online Mendelian Inheritance in Man (OMIM, https://www.omim.org/ (accessed on 18 January 2026)), Human Gene Mutation Database (HGMD, http://www.hgmd.cf.ac.uk (accessed on 18 January 2026)), Clinical Genome Resource (ClinGen, https://clinicalgenome.org/ (accessed on 18 January 2026)), Genomics England PanelApp (PanelApp, https://panelapp.genomicsengland.co.uk/ (accessed on 18 January 2026)), U.S. National Library of Medicine (PubMed, https://pubmed.ncbi.nlm.nih.gov/ (accessed on 18 January 2026)). Whole exome sequencing was applied as a second-line test in patients (P24, P56, P69, P76) who had a negative result (no disease causing variant detected) from the multi-gene panel and in whom a definitive molecular diagnosis was still sought. WES was also used as a first-line test in patients (P08, P35, P52, P53, P70, P72, P80) presenting with multiple manifestations, including neurodevelopmental disorders or atypical phenotype (as described chapter 4 of the Discussion).

Raw FASTQ reads were mapped to a slightly modified human genome assembly GRCh38/hg38. Variant calling was performed using GATK HaplotypeCaller, MuTect2, FreeBayes, and DeepVariant to maximize sensitivity for detecting single-nucleotide variants (SNVs), small insertions/deletions, and mosaic variants. Copy-number variant (CNV) analysis was conducted with CNVkit and Decon. Detailed information on the methodology has been published previously by P. Halat-Wolska et al. [7]. Alignments were visualized using the Integrative Genomics Viewer (IGV, https://igv.org/ (accessed on 18 January 2026)) [8].

Variant filtering criteria included allele frequency, in silico pathogenicity predictions, and the predicted effect and genomic location of the variant. Variant consequence annotation was performed using VEP [9]. Variants were further annotated using multiple repositories, including population frequency databases such as the Genome Aggregation Database (gnomAD v4.1.0, https://gnomad.broadinstitute.org (accessed on 18 January 2026)), and an in-house allele-frequency database representing the Polish population (POLdb), which contains data from more than 24,000 individuals suspected of having a rare genetic disease. Predicted impact on protein structure and function was assessed using machine learning meta-predictors (BayesDel, REVEL) and individual tools (AlphaMissense, CADD, EIGEN, FATHMM-MKL, MutationTaster, PolyPhen-2, SIFT). Variants occurring at splice sites were analyzed using SpliceAI, ADA, MaxEntScan, Pangolin and RF. In silico predictions were retrieved through the GeneBe (https://genebe.net/ (accessed on 18 January 2026)) and VarSome platforms (https://varsome.com/ (accessed on 18 January 2026)). Alamut Visual^TM^ Plus software (https://www.sophiagenetics.com/platform/alamut-visual-plus/ (accessed on 18 January 2026)) was also used. Conservation was assessed using phyloP100 scores. The novelty of detected variants was evaluated using ClinVar (https://www.ncbi.nlm.nih.gov/clinvar (accessed on 18 January 2026)), the Leiden Open Variation Database (LOVD, https://www.lovd.nl/ (accessed on 18 January 2026)), and the Human Gene Mutation Database (HGMD, http://www.hgmd.cf.ac.uk (accessed on 18 January 2026)).

Variants were interpreted according to the guidelines of the American College of Medical Genetics and Genomics and the Association for Molecular Pathology (ACMG/AMP) [7,10,11]. ACMG/AMP criteria were assigned using the GeneBe platform [12], followed by manual refinement of criteria that could not be processed automatically to ensure accurate pathogenicity classification. According to these criteria, benign and likely benign variants were excluded, and only pathogenic and likely pathogenic (P/LP) variants were retained for analysis. The present study summarizes the results of probands with identified P/LP variants. Patients harboring variants of uncertain significance (VUS) will undergo more detailed clinical and genetic evaluation in the coming years.

#### 2.2.2. Sanger Sequencing

Variants considered (potentially) disease-causing were validated in probands and, when possible, segregated in available family members using bidirectional Sanger sequencing with the BigDye Terminator v3.1 Kit (Applied Biosystems, Waltham, MA, USA) on the ABI 3130 Genetic Analyzer (Applied Biosystems, Waltham, MA, USA), according to the manufacturer’s protocol. The nomenclature of molecular variants followed the Human Genome Variation Society (HGVS) guidelines (https://hgvs-nomenclature.org/ (accessed on 18 January 2026)) and was referenced to the MANE Select transcript [13], which is aligned with the GRCh38/hg38 human genome assembly.

## 3. Results

### 3.1. Clinical Characteristics of Patients

The study cohort consisted of 87 probands: 42 males (48.3%) and 45 females (51.7%) with epilepsy, developmental and epileptic encephalopathies (DEE), early-onset epilepsies, or syndromic neurodevelopmental disorders. Detailed clinical characteristics of the cohort are presented in Table 1.

In 45 cases, epilepsy began in the neonatal or early-infantile period, presenting with tonic–clonic, myoclonic, tonic, clonic, atonic, or febrile seizures, absence episodes, or infantile spasms (West syndrome). Generalized seizures were observed in 28 patients, while 11 experienced focal or multifocal seizures. Additionally, 5 patients had both generalized and focal seizures, in 48 patients the seizure type could not be determined. Global developmental delay or intellectual disability was present in 41 patients, motor disability in 33, and speech delay in 23. EEG abnormalities—such as epileptiform discharges, hypsarrhythmia, suppression–burst patterns, or other atypical findings—were documented in 36 patients. Drug-resistant seizures were noted in 27 individuals. MRI abnormalities were identified in 21 patients and included structural brain malformations (e.g., cerebellar hypoplasia, holoprosencephaly, polymicrogyria, schizencephaly), delayed myelination, and progressive cerebral atrophy. Epileptic seizures occurred as part of various genetic syndromes with dysmorphic features in 17 patients.

**Figure 1:**
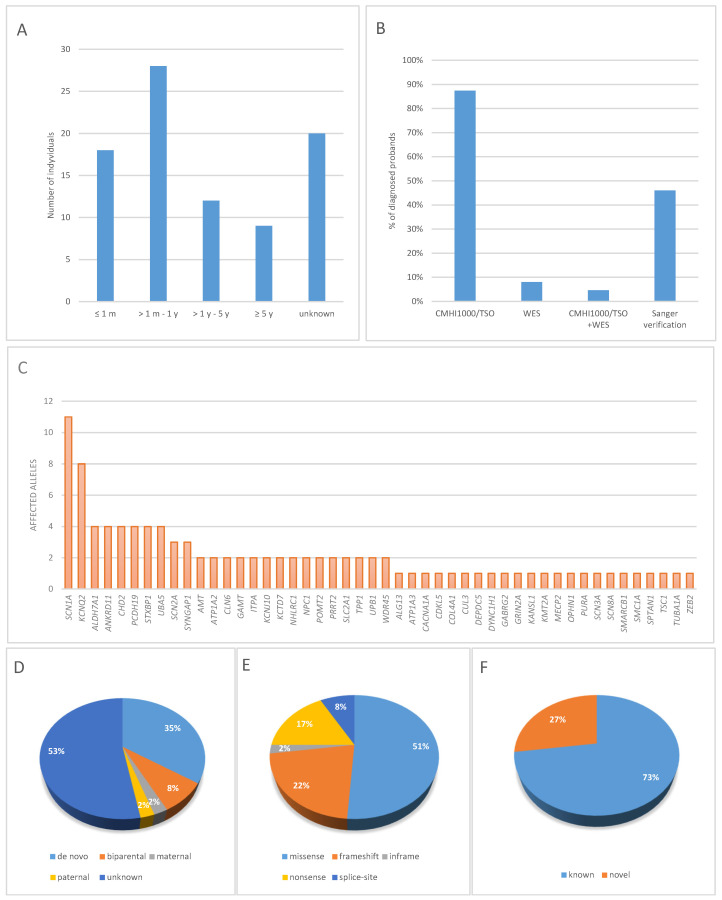

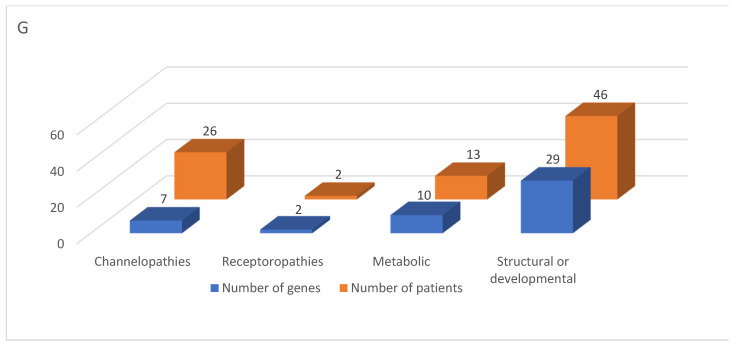
Proportion of diagnoses achieved through NGS-based genetic testing: (**A**) Differences in the rate of genetic diagnoses by age of onset, (**B**) type of genetic test, (**C**) The contribution of genes to the etiology of epilepsy in the studied cohort, (**D**) Inheritance patterns, (**E**) Types of pathogenic or likely pathogenic variants, (**F**) Proportion of known and novel molecular variants, (**G**) Genetic etiology profile of epilepsy in pediatric patients.

Neonatal-onset seizures were observed in 18 patients (20.7%). In most patients (*n* = 28), the first seizure episode occurred between 1 month and 1 year of age; in 12 patients between 1 and 5 years; and in 9 patients at ≥5 years of age. In 20 patients, seizure onset could not be determined (Figure 1A).

### 3.2. Molecular Findings and Genotyping

Among 87 patients tested with next-generation sequencing, 76 (87.4%) had only NGS panel (CMHI1000 or TSO) performed, and 7 (8%) underwent only whole exome sequencing. In 4 patients with negative result of panel sequencing, the analysis was expanded to whole exome (Table 1, Figure 1B). Sanger sequencing was used to verify variants in 40 probands (46%) (Figure 1B). Qualifying variants identified in the study group are listed in Supplementary Table S3.

The molecular profile of the study cohort is presented in Figure 1C. In total, 88 different variants were detected in 48 genes, of which variants affecting 10 genes (*SCN1A*, *KCNQ2*, *ALDH7A1*, *ANKRD11*, *CHD2*, *PCDH19*, *SCN2A*, *STXBP1*, *SYNGAP1*, *UBA5*) accounted for 48% (49/102) of all variants identified (Supplementary Table S4, Figure 1C). In addition, single individuals had variants identified in the following genes: *ALG13*, *AMT*, *ATP1A3*, *CACNA1A*, *CDKL5*, *CLN6*, *COL4A1*, *CUL3*, *DEPDC5*, *DYNC1H1*, *GABRG2*, *GAMT*, *GRIN2A*, *ITPA*, *KANSL1*, *KCNJ10*, *KCTD7*, *KMT2A*, *MECP2*, *NHLRC1*, *NPC1*, *OPHN1*, *POMT2*, *PURA*, *SCN3A*, *SCN8A*, *SMARCB1*, *SMC1A*, *SPTAN1*, *TPP1*, *TSC1*, *TUBA1A*, *UPB1*, and *ZEB2*. The causative genes in patients with neonatal-onset epilepsy included *ALDH7A1*, *AMT*, *ATP1A3*, *COL4A1*, *GRIN2A*, *KCNQ2*, *PURA*, *SCN2A*, *SCN3A*, and *STXBP1*, whereas in patients with seizure onset after 5 years of age, the causative genes included *CACNA1A*, *DEPDC5*, *CLN6*, *DYNC1H1*, *NHLRC1*, *NPC1*, *SLC2A1*, *STXBP1*, and *ZEB2*.

Among all detected variants, pathogenic or likely pathogenic single-nucleotide variants (SNVs) were found in 87 patients. There were 45 missense variants, 15 nonsense variants, 11 frameshift deletions, 7 frameshift duplications, 7 splicing alterations, and single occurrences of a frameshift indel, an in-frame deletion, and an in-frame duplication (Figure 1E).

Family segregation was carried out in 41 probands, identifying two parents with symptomatic autosomal dominant epilepsy (P07, P40). Autosomal dominant (AD) conditions were identified in 70.1% of patients (61/87). In this group, 28.7% (25/87) had de novo pathogenic or likely pathogenic (P/LP) variants, while 2.3% (2/87) inherited variants from an affected heterozygous parent (Table 1). Autosomal recessive (AR) conditions were diagnosed in 15 patients (15/87). X-linked (XL) conditions were identified in 12.6% (11/87), including 4 de novo variants, 1 hemizygous variant in *OPHN1* inherited from an unaffected heterozygous mother, and 1 heterozygous variant in *PCDH19* inherited from an unaffected hemizygous father, consistent with the specific X-linked inheritance pattern. In total, among all 88 variants identified, 12% were of parental origin, whereas 35% occurred de novo. In 46 cases (53%), segregation studies could not be performed (Figure 1D). In these instances, parental samples were not available, either because the families did not present for sample collection, exercised their right to refuse genetic testing or attempts to contact them were unsuccessful.

Among the identified variants, 64 had been previously reported in databases such as ClinVar, HGMD, or LOVD, while 24 were novel (Figure 1F). According to ACMG/AMP criteria, 18 variants were classified as pathogenic (P) and 6 as likely pathogenic (LP). Sixteen of the newly identified variants were loss-of-function (LoF) and thus met the PVS1 criterion. Eight variants were missense changes, five of which occurred de novo (PM6). For one missense variant, c.3789C>A in *SCN1A* (P03), segregation analysis in the parents was not performed. However, within this hotspot region, eight amino acids have pathogenic missense changes in a ±8 amino-acid window around the variant, and another nucleotide change resulting in the same amino acid substitution has previously been reported as likely pathogenic in ClinVar, supporting criteria PS1, PM1, and PM2.

We identified 11 novel molecular variants classified as P/LP according to the ACMG criteria: *ANKRD11*: c.1442C>A and c.6191_6192del, *CHD2*: c.4797_4798dupCA, *COL4A1*: c.4031G>A, *PURA*: c.885delinsGC, *SCN1A*: c.752T>A and c.2468A>T, *SCN8A*: c.2895C>A, *SMARCB1*: c.919T>C, *SMC1A*: c.3061dup, *WDR45*: c.103G>T (Supplementary Table S3). Five variants were recurrent: *ALDH7A1*: c.328C>T, *KCNQ2*: c.1678C>T, *PRRT2*: c.649dup, *STXBP1*: c.1162C>T, *UBA5*: c.796G>C; each identified in two unrelated probands. Given this limited number of observations, it is not possible to reliably assess a potential founder effect. These variants should therefore be regarded as rare within our population, and the available data do not support a hypothesis of shared ancestry.

The ACMG/AMP classification for each individual variant is provided in the publicly available original GeneBe database and is also summarized in the column “Variant ACMG classifications” in Supplementary Table S3.

Analysis of the molecular background in our cohort enabled classification of epilepsies into four categories based on genetic pathomechanisms: channelopathies (variants in 7 genes; 26 patients), receptoropathies (variants in 2 genes; 2 patients), metabolic epilepsies (variants in 10 genes; 13 patients), and structural-genetic/developmental epilepsies (variants in 29 genes; 46 patients) (Table 2).

## 4. Discussion

Childhood and early-infantile epilepsy are characterized by marked genetic and molecular heterogeneity. In recent years, high-throughput technologies such as exome sequencing have revealed a large number of genes implicated in epileptogenesis, many of which contribute to extremely rare, individualized phenotypes. This molecular diversity limits the reliability of classification based solely on clinical presentation and necessitates a diagnostic approach focused on identifying the specific underlying genetic cause. In our study group, the identified variants correlated with the symptoms observed in probands. The majority of patients had concomitant neurodevelopmental disorders, which were frequent in patients with developmental and epileptic encephalopathy (DEE) or epilepsy with intellectual disability/developmental delay (ID/DD) (48%, 42/87 and 47%, 41/87, respectively).

The most common type of seizure was generalized, observed in 32% (28/87) of patients, whereas nocturnal seizures were detected in 3.5% (3/87). However, in many cases, it was difficult to determine the type of seizure, and therefore the type of epilepsy was not classified in 24 patients (27.6%). Refractory seizures were observed in 31% (27/87) of patients. MRI abnormalities were detected in 24.1% (21/87) of patients, although the performance of MRI scans was documented in only 47% (41/87) of cases. According to current guidelines, brain imaging is recommended for all children with epilepsy [14]. EEG abnormalities were present in 41.4% (36/87) of patients. Additionally, epilepsy associated with metabolic disorders was detected in 13 patients (14.9%). In 42 patients, a diagnosis of DEE was confirmed through genetic testing. The most frequent causative gene, found in 12.6% (11/87) of patients, was *SCN1A*, known to be associated with both DEE and Dravet syndrome. The diagnostic yield was highest in patients with a more severe disease course, earlier age of onset, DEE diagnosis, or concomitant intellectual disability or motor impairment [15,16,17]. According to previous studies, the diagnostic yield for DEE is expected to reach 32% [15]. Similar results were obtained in another Polish study on the genetic background of epilepsy [18], in which the highest rate of positive diagnoses applied to children with ID/DD and generalized or generalized/focal onset seizures (41%) and in children with DEEs (40%). These results indicate that ID/DD is the main factor influencing the percentage of positive epilepsy diagnoses.

It should be emphasized that an accurate assessment of diagnostic yield requires the inclusion of all tested individuals, including patients with both positive and negative results. In the present study, diagnostic yield was therefore not evaluated, as the analysis was limited to patients harboring pathogenic or likely pathogenic (P/LP) variants, while cases with negative or inconclusive results, including variants of uncertain significance (VUS), were excluded. Consequently, the findings presented here should not be interpreted as a measure of diagnostic yield, but rather as an indication of the utility of the applied gene panels in identifying pathogenic variants associated with developmental and epileptic encephalopathies (DEE).

In 11 patients with clinically suspected Dravet syndrome (DS), a confirmatory pathogenic or likely pathogenic variant in the *SCN1A* gene was identified in 7 patients. The remaining 4 patients had variants in the following genes: *ATP1A3* (associated with developmental and epileptic encephalopathy 99), *KMT2A* (associated with Wiedemann-Steiner syndrome), *PCDH19* (associated with developmental and epileptic encephalopathy 9), and *SYNGAP1* (associated with intellectual developmental disorder, autosomal dominant 5). This indicates that an initial clinical diagnosis of Dravet syndrome is not always associated with *SCN1A* gene alterations. Dravet syndrome may initially manifest as febrile seizures; while 70–80% of patients with DS have *SCN1A* loss-of-function (LoF) variants, Dravet-like phenotypes can also result from pathogenic variants in other genes, including *CHD2*, *GABRA1*, *GABRG2*, *SCN1B*, *SCN2A*, *SCN8A*, *PCDH19*, and *STXBP1* [19]. In a study by JM de Sainte Agathe, 29% of individuals carrying *SCN1A* LoF variants had not been clinically classified as having DS, which was an unexpected finding. In that cohort, 58% of patients were below 2 years old at the time of first presentation, rendering a diagnosis of intellectual disability premature. Conversely, several patients with a clinical diagnosis of DS were ultimately found to carry pathogenic variants in genes more frequently associated with other phenotypes. This likely reflects the high prevalence of febrile seizures outside the Dravet syndrome/generalized epilepsy with febrile seizures plus (GEFS+) spectrum. Notably, febrile triggers were observed in 30% of DEE cases, while the DS spectrum accounted for only 10% of the DEE cohort [20].

Among four patients with atypical clinical presentations of DEE, patient P06 had a homozygous variant c.520T>C p.(Trp174Arg) in the *GAMT* gene, typically associated with cerebral creatine deficiency syndrome 2 (MIM #612736); patient P24 had a de novo hemizygous variant c.103G>T p.(Glu35*) in *WDR45*, associated with neurodegeneration with brain iron accumulation 5 (MIM #300894); patient P81 had a homozygous variant c.179T>C p.(Ile60Thr) in *KCNJ10*, associated with SESAME syndrome (MIM #612780); and patient P83 carried a novel de novo variant c.885delinsGC p.(His296Profs*21) in *PURA*, associated with neurodevelopmental disorder with neonatal respiratory insufficiency, hypotonia, and feeding difficulties (MIM #616158). Since variants in these genes are not typical causes of DEE, these cases highlight the difficulty in distinguishing seizure-accompanied ID or developmental delay from true DEE, in which ID results partly from uncontrolled epileptic activity [21].

In nine other patients with an unidentified epilepsy phenotype, genetic testing enabled the diagnosis of known syndromic genetic disorders associated with the following genes: *ANKRD11* (KBG syndrome) in four patients, *KANSL1* (Koolen-De Vries syndrome), *KMT2A* (Wiedemann-Steiner syndrome), *MECP2* (Rett syndrome), *OPHN1* (X-linked syndromic intellectual developmental disorder, Billuart type), *SMARCB1* (Coffin-Siris syndrome 3), and *ZEB2* (Mowat–Wilson syndrome). Syndromic epilepsies are characterized by substantial phenotypic heterogeneity, including dysmorphic features, multisystem involvement, and neurodevelopmental impairments. Consequently, traditional clinical criteria used to differentiate these conditions have limited specificity and stability, particularly in children aged 1–6 years. The clinical presentation of many syndromic forms evolves over time, further reducing the reliability of phenotype-based classification. In these patients, a “genotype-first” diagnostic approach has proven particularly valuable.

Four patients underwent multiple genetic tests and received a diagnosis only after WES. The molecular changes involved the following genes: *UBA5*, *UPB1*, and *WDR45*. Using WES as a first-line diagnostic tool could allow molecular diagnoses in some cases. For example, patients P56 and P76, who presented with epilepsy and severe neurological symptoms, were found to carry variants in *UBA5* following a negative CMHI1000 panel result: a novel c.796G>C p.(Val266Leu) variant, and two other variants in trans: a novel c.698T>A p.(Leu233His) and a known c.1111G>A p.(Ala371Thr). The *UBA5* gene is associated with developmental and epileptic encephalopathy 44. Previous Polish studies confirm the high effectiveness of dedicated epilepsy panels [18]. According to the National Society of Genetic Counselors, multi-gene panels should include a minimum of 25 genes [22]. Literature suggests that a “basic” panel of 30 genes accounts for most diagnoses, while larger panels contribute only marginally to additional findings. Moreover, evaluating the diagnostic yield of genetic tests requires careful cohort selection (e.g., childhood vs. adult epilepsy, focal epilepsy, DEE, metabolic or structural epilepsy) [23].

In the study by Krygier et al., only 5 of 127 patients were diagnosed using WES, while 41 were diagnosed using a gene panel [18]. Of 16 patients who underwent WES after a negative CMHI1000/TSO panel, only one yielded a molecular diagnosis. Krey et al. highlighted the potential for precision therapy based on variants in genes: *ALDH7A1*, *PNPO*, *PLPBP*, *CAD*, *CHRNA2*, *CHRNA4*, *CHRNB2*, *GRIN2A*, *GRIN2B*, *GRIN2D*, *KCNA2*, *KCNQ2*, *KCNQ3*, *SCN1A*, *SCN2A*, *SCN8A*, *SLC2A1*, *TSC1*, *TSC2* [24]. Fifteen of these genes are included in the CMHI1000 panel used in our study.

Interestingly, among four patients with *PCDH19* variants, one male patient (P59) presented with DEE and Klinefelter syndrome (47, XXY). To date, only one patient with this combination has been reported [25]. *PCDH19*-clustering epilepsy (PCDH19-CE) is an X-linked DEE characterized by clusters of focal or generalized seizures, typically beginning between 6 and 36 months of age, often triggered by febrile or afebrile illnesses, and associated with a wide spectrum of neuropsychiatric features. The “cellular interference” mechanism explains why heterozygous females and mosaic males are affected, while hemizygous males are usually spared, resulting in considerable phenotypic variability that does not consistently correlate with variant type [25,26,27,28,29,30,31,32,33,34,35]. In our cohort, we describe four patients with PCDH19-CE, including one boy with Klinefelter syndrome, who had drug-resistant febrile and generalized tonic–clonic seizures starting at 2.5 years, psychomotor and speech delay, and normal MRI findings. A heterozygous c.2452C>T p.(Gln818*) variant in *PCDH19* was identified. Three additional female patients (P32, P43, P44) exhibited variable phenotypes, ranging from drug-resistant early-onset seizures with developmental delay to milder epilepsy without cognitive impairment. These cases highlight that disease severity is influenced more by mosaicism and age at seizure onset than by variant type, emphasizing the challenges of predicting outcomes based solely on molecular findings.

Genotype–phenotype correlations suggest that objective clinical data may be more predictive than classification into specific epilepsy syndromes [20]. Thorough phenotypic characterization, including MRI and biochemical tests, is critical. In patients with seizure onset before one year of age, who constituted the majority of our cohort, the phenotype may be incomplete, and early genetic diagnosis could enable targeted therapy. Our findings confirm that clinical symptoms and genetic etiology do not always align.

Among epilepsies with a confirmed genetic basis, many are monogenic disorders with autosomal dominant inheritance; however, a substantial proportion of variants arise de novo [36]. Metabolic epilepsies are most frequently associated with autosomal recessive defects [37]. In our study, autosomal dominant conditions were identified in 70.1% of patients, autosomal recessive in 17.2%, and X-linked in 12.6%. This is broadly consistent with Lindy et al., where ~75% of positive cases carried P/LP variants in AD genes and 18.2% in XL genes [38], though our cohort showed a higher proportion of AR inheritance. Missense variants were the most common type of SNV (51%), followed by nonsense (17%), frameshift deletions (13%), frameshift duplications (8%), splicing alterations (8%), frameshift indels (1%), in-frame deletions (1%), and in-frame duplications (1%) (Figure 1E). We identified 24 novel variants, including 16 LoF (66.7%) and 8 missense variants (33.3%).

Consistent with numerous studies across diverse populations, our data confirm that, despite substantial genetic heterogeneity, a limited number of genes account for a large proportion of pediatric epilepsy cases. Large cohort and gene panel–based studies from Europe, North America, and Asia have repeatedly demonstrated that a small group of genes dominates the etiology of pediatric epilepsies. Frequently implicated genes include *SCN1A*, *SCN2A*, *KCNQ2*, *STXBP1*, *PCDH19*, and *CDKL5*, as well as several genes encoding GABA receptor subunits. Together, these genes account for a substantial proportion of early-onset epileptic encephalopathies and primary channelopathies, making them key diagnostic targets [15,18,38,39,40,41,42,43].

In our cohort, 48% of molecular diagnoses involved the ten most frequently identified genes: *SCN1A* (n = 11), *KCNQ2* (n = 8), *ALDH7A1* (n = 4), *ANKRD11* (n = 4), *CHD2* (n = 4), *PCDH19* (n = 4), *STXBP1* (n = 4), *UBA5* (n = 4), *SCN2A* (n = 3), and *SYNGAP1* (n = 3), which the numbers in parentheses indicate the number of affected alleles identified for each gene. This distribution reflects the predominant contribution of sodium channel and synaptic genes reported in population-based studies from both Western and Asian cohorts [38,43,44]. Notably, in the study by Lindy et al., more than 80% of diagnoses were attributable to the seven most frequently implicated genes [38].

The recurrent identification of *PCDH19, CHD2*, *SYNGAP1*, and *STXBP1* in our cohort is consistent with findings from large diagnostic series demonstrating their significant contribution to developmental and epileptic encephalopathies [38,45,46]. Similar gene distributions have been reported in other studies, including *ANKRD11*, *CHD2*, *PCDH19*, and *STXBP1* [15], as well as *SCN1A*, *KCNQ2*, and *PCDH19* [47]. The prominence of *SCN1A* variants in our patients aligns with previous pediatric studies identifying this gene as the most common cause of genetic epilepsies, particularly Dravet syndrome and related phenotypes [6,15,38,39]. In the study by Krygier et al., *PCDH19* and *STXBP1* were among the most frequently identified genes alongside *SCN1A* [18], whereas *ALDH7A1* and *KCNQ2* were also prominent in the cohort reported by Andjelkovic et al. [6]. The presence of *ALDH7A1* among the more frequently identified genes in our cohort further aligns with reports highlighting its role in early-onset and potentially treatable epilepsies across different populations [18,48].

Most identified variants were unique. Only eight genes were detected in more than two individuals. However, six recurrent variants were observed among unrelated probands, including c.649dupC in *PRRT2*, which has also been reported as the most common recurrent variant in multiple independent studies [20,38,49]. Despite these recurrent variants, none were dominant, reflecting the high genetic variability characteristic of childhood epilepsy. This pattern aligns with observations from multiple populations [50,51,52].

Overall, the genetic architecture observed in the Polish cohort closely parallels that reported in global population-based studies. Minor differences in gene frequencies are likely attributable to cohort composition, age at seizure onset, and referral patterns rather than population-specific effects. These findings underscore that established epilepsy-associated genes constitute robust diagnostic targets across diverse populations. Despite these recurrent variants, none were dominant, reflecting the high genetic variability characteristic of childhood epilepsy. This pattern aligns with observations from multiple populations [50,51,52]. Our data confirm that epilepsy is a multigenic and multifactorial disorder, where a single epileptic syndrome may involve multiple pathogenic events and may be classified into more than one seizure type or etiological category. Consequently, it is reasonable to classify epilepsy based on the molecular pathomechanism of the causative gene. In this study, genes were categorized according to the function of the encoded protein: channelopathies, receptoropathies, metabolic, and structural/developmental. The largest group comprised structural/developmental genes (29 genes in 52.9% of patients). Ion channel gene variants represented a substantial functional category, affecting 29.9% of patients, although they accounted for only seven genes (14.6%). By comparison, Liu et al. reported ion channel gene variants as the largest functional category (55.8%) [53] while Henry O. et al. found ion channel genes as the most enriched group (24.8%) [49]. Our study confirms the high incidence of epilepsy during the first year of life (53%), consistent with previous reports [54]. Stodberg T et al. demonstrated that two-thirds of children with epilepsy onset in the first year of life can receive a specific diagnosis [14].

Next-generation sequencing (NGS) enabled molecular diagnoses regardless of structural or metabolic findings, substantially increasing diagnostic yield [14]. In our cohort, 68% of patients with known genetic etiologies had structural or metabolic abnormalities, a proportion higher than in previous studies. No significant differences were observed between patients with onset in the first year of life and those with later onset regarding the prevalence of structural/metabolic genetic disorders. A recent meta-analysis by Sheidley et al. (2022) reported a 19% diagnostic yield for multi-gene panels in epilepsy, with the highest yield observed in patients with DEE [23].

Genetic testing is essential in evaluating epilepsy, particularly in early-onset, severe, or treatment-resistant cases. Identifying a pathogenic variant provides a precise diagnosis, guiding prognosis, and potentially reducing the need for invasive or costly tests. A confirmed genetic result also informs counseling by clarifying inheritance patterns, recurrence risk, family planning and identifying at-risk relatives. Importantly, genetic findings increasingly guide therapy. Advances in epilepsy genetics have enabled a shift from empiric antiseizure medication selection (ASM) toward genetically driven and precision-based therapies. Genetic variation influences ASM efficacy and tolerability through effects on pharmacokinetics, pharmacodynamics, and adverse drug reactions, supporting the clinical utility of pharmacogenomics in epilepsy management [55]. Importantly, pathogenic variants in epilepsy-associated genes can directly guide drug choice. For example, gain-of-function mutations in sodium channel genes such as *SCN2A* or *SCN8A* may respond favorably to sodium channel blockers, whereas loss-of-function variants and *SCN1A*-related epilepsies often show poor response or seizure worsening with these agents, highlighting the need for genotype-guided pharmacotherapy [56]. Beyond tailored pharmacological treatment, gene-based therapies aim to address the underlying molecular cause of epilepsy. Gene therapy approaches include gene replacement, gene silencing, and modulation of neuronal excitability, most commonly using adeno-associated viral (AAV) vectors [57]. While these approaches hold considerable promise, challenges related to delivery, specificity, timing, and long-term safety remain, and continued translational research is required before widespread clinical implementation [58].

## 5. Conclusions

1. Our study confirms that next-generation sequencing (NGS), including both targeted gene panels and whole-exome sequencing (WES), is an effective and reliable diagnostic tool for pediatric epilepsy. These approaches substantially improve the detection of underlying genetic causes, facilitating more accurate clinical decision-making and enabling individualized therapeutic strategies.
2. Establishing an etiological diagnosis remains essential for accurate disease classification and for guiding both current and future pharmacological interventions.
3. The identification of novel genetic markers—both genes and variants—and their associated phenotypes expands the clinical and genetic spectrum of epilepsies, providing valuable insights into the pathomechanism of these disorders in children.
4. The molecular profile observed in our Polish pediatric cohort closely mirrors patterns reported in the literature, supporting the broader applicability and generalizability of our findings.

## Figures and Tables

**Table 1:** Clinical characteristics and genotyping of 87 individuals.

Patient ID	Gender	Epileptic Encephalopathy Suspected	Dravet Syndrome Suspected	Generalized/Focal Seizures	Type of Seizures	Seizures Onset	Dysmorphism	ID/Developmental Delay or Regression	Motor Delay	Speech Delay	Refractory Seizures	MRI Abnormalities	EEG Abnormalities	Genetic Testing Method	Gene	Genotype (cDNA)	Protein Effect	hereditary Pattern	Type of Inheritance	Genotype-Fenotype Correlation [OMIM]	Classification of Epilepsies Based on Genetic/Molecular Pathomechanism
P01	M			n/a	n/a	n/a	n/a	yes	yes	n/a	n/a	yes	n/a	TSO, Sanger	*TUBA1A*	c.[920C>T];[=]	p.[Pro307Leu];[=]	de novo	AD	LIS3	DEVELOP
P02	M	yes		n/a	TON	4 m	n/a	n/a	n/a	yes	n/a	yes	yes	TSO, Sanger	*ITPA*	c.[124+1G>A];[124+1G>A]	p.[?];[?]	biparental	AR	DEE35	DEVELOP
P03	F	yes	yes	GE	MYOCLON	6 m	n/a	n/a	n/a	n/a	yes	n/a	n/a	TSO, Sanger	*SCN1A*	c.[3789C>A];[=]	p.[Phe1263Leu];[=]	n/a	AD	DS/DEE6B/GEFSP2	CHANNNEL
P04	F	yes		n/a	TON-CLON	1 d	n/a	n/a	n/a	n/a	yes	n/a	yes	CMHI1000, Sanger	*KCNQ2*	c.[802C>T];[=]	p.[Leu268Phe];[=]	de novo	AD	DEE7	CHANNNEL
P05	F			GE	n/a	2 m	n/a	yes	yes	n/a	n/a	yes	n/a	CMHI1000, Sanger	*CDKL5*	c.[1675C>T];[=]	p.[Arg559*];[=]	de novo	XLD	DEE2	DEVELOP
P06	F	yes		n/a	TON-CLON, FEBR, ATON	n/a	n/a	yes	n/a	n/a	yes	yes	yes	CMHI1000	*GAMT*	c.[520T>C];[(520T>C)]	p.[Trp174Arg];[=]	n/a	AR	CCDS2	METAB
P07	M		yes	GE	FEBR	6 m	n/a	n/a	n/a	n/a	n/a	n/a	n/a	CMHI1000, Sanger	*SCN1A*	c.[2576G>A];[=]	p.[Arg859His];[=]	maternal	AD	DS	CHANNNEL
P08	F	yes		FE	IS, TON-CLON	5 d	n/a	yes	yes	n/a	yes	n/a	yes	WES, Sanger	*GRIN2A*	c.[1903G>A];[=]	p.[Ala635Thr];[=]	de novo	AD	FESD	RECEPTOR
P09	F		yes	GE	ABSEN	8 m	n/a	no	no	n/a	n/a	n/a	n/a	CMHI1000, Sanger	*SCN1A*	c.[752T>A];[=]	p.[Met251Lys];[=]	de novo	AD	DS	CHANNNEL
P10	F	yes		GE	ABSEN, NOC, TON-CLON, MYOCLON	n/a	n/a	yes	n/a	yes	n/a	no	yes	CMHI1000, Sanger	*CHD2*	c.[4797_4798dupCA];[=]	p.[Asn1600Thrfs*215];[=]	de novo	AD	DEE94	DEVELOP
P11	M			n/a	IS, TON, TON-CLON	1 d	yes	n/a	n/a	n/a	yes	yes	yes	CMHI1000, Sanger	*SCN3A*	c.[3998C>T];[=]	p.[Pro1333Leu];[=]	de novo	AD	DEE62	CHANNNEL
P12	M		yes	GE	FEBR, TON-CLON	8 m	n/a	no	no	no	n/a	n/a	n/a	CMHI1000	*SCN1A*	c.[1177C>T];[=]	p.[Arg393Cys];[=]	n/a	AD	DS	CHANNNEL
P13	F			FE	TON, CLON	2 m	n/a	yes	yes	n/a	n/a	n/a	yes	CMHI1000, Sanger	*STXBP1*	c.[1162C>T];[=]	p.[Arg388*];[=]	de novo	AD	DEE4	DEVELOP
P14	F			n/a	IS, MYOCLON	5 m	n/a	yes	yes	n/a	yes	no	yes	TSO	*ALG13*	c.[320A>G];[=]	p.[Asn107Ser];[=]	n/a	XL	DEE36 (WS)	DEVELOP
P15	F		yes	n/a	CLON	3 y	yes	yes	yes	n/a	n/a	n/a	yes	CMHI1000, Sanger	*SYNGAP1*	c.[333del];[=]	p.[Lys114Serfs*20];[=]	de novo	AD	MRD5	DEVELOP
P16	M			n/a	IS, CLON	1 m	n/a	n/a	n/a	n/a	n/a	yes	n/a	TSO, Sanger	*COL4A1*	c.[4031G>A];[=]	p.[Gly1344Asp];[=]	de novo	AD	BSVD1	DEVELOP
P17	M			n/a	n/a	n/a	yes	n/a	n/a	n/a	n/a	n/a	n/a	CMHI1000, Sanger	*KANSL1*	c.[808_809del];[=]	p.[Leu270Valfs*11];[=]	de novo	AD	KDVS	DEVELOP
P18	M			n/a	n/a	n/a	no	yes	yes	yes	yes	n/a	n/a	CMHI1000	*SYNGAP1*	c.[509G>A];[=]	p.[Pro170Argfs*25];[=]	n/a	AD	MRD5	DEVELOP
P19	F		yes	n/a	FEBR, TON-CLON, ABSEN	6 m	n/a	n/a	yes	n/a	yes	no	no	CMHI1000	*SCN1A*	c.[301C>T];[=]	p.[Arg101Trp];[=]	n/a	AD	DS	CHANNNEL
P20	M	yes		MFE	MYOCLON, IS	4 m	yes	yes	yes	n/a	yes	yes	yes	CMHI1000, Sanger	*SPTAN1*	c.[6908_6916dup];[=]	p.[Asp2303_Leu2305dup];[=]	de novo	AD	DEE5	DEVELOP
P21	F			GE	n/a	13 y	n/a	yes	n/a	n/a	n/a	n/a	yes	CMHI1000	*CACNA1A*	c.[4252C>T];[=]	p.[Arg418*];[=]	n/a	AD	DEE42	CHANNNEL
P22	M	yes	yes	n/a	CLON, MYOCLON, NOC, ATON	2 d	n/a	n/a	n/a	n/a	n/a	n/a	n/a	CMHI1000	*ATP1A3*	c.[2443G>A];[=]	p.[Glu815Lys];[=]	n/a	AD	DEE99	DEVELOP
P23	M			GE	ATON, MYOCLON	n/a	yes	yes	n/a	n/a	n/a	no	n/a	CMHI1000, Sanger	*ANKRD11*	c.[1442C>A];[=]	p.[Ser481*];[=]	de novo	AD	KBGS	DEVELOP
P24	M	yes		GE	CLON, MYOCLON	10 m	n/a	yes	yes	n/a	yes	yes	yes	CMHI1000, WES	*WDR45*	c.[103G>T];[0]	p.[Glu35*];[0]	de novo	XLD	NBIA5	METAB
P25	M			n/a	n/a	n/a	yes	n/a	n/a	n/a	n/a	yes	n/a	TSO, Sanger	*POMT2*	c.[70del];[70del]	p.[Gln24Argfs*41];[Gln24Argfs*41]	biparental	AR	MDDGA2	DEVELOP
P26	F			n/a	MULTIPLE	n/a	n/a	n/a	n/a	n/a	n/a	n/a	n/a	CMHI1000	*SCN1A*	c.[302G>A];[=]	p.[Arg101Gln];[=]	n/a	AD	DS/DEE6B/GEFSP2	CHANNNEL
P27	F			n/a	n/a	n/a	n/a	yes	yes	n/a	yes	n/a	n/a	CMHI1000	*TPP1*	c.[622C>T(;)833A>C]	p.[Arg208*(;)Gln278Pro]	n/a	AR	CLN2	METAB
P28	M	yes		GE, FE	FEBR	18 m	n/a	n/a	n/a	n/a	n/a	no	n/a	CMHI1000, Sanger	*SCN1A*	c.[4219C>T];[=]	p.[Arg1407*];[=]	de novo	AD	DS/DEE6B/GEFSP2	CHANNNEL
P29	M	yes		FE	n/a	n/a	n/a	n/a	n/a	n/a	n/a	n/a	n/a	CMHI1000	*SCN2A*	c.[781G>T];[=]	p.[Val261Leu];[=]	n/a	AD	DEE11	CHANNNEL
P30	F	yes		n/a	n/a	2 m	n/a	n/a	n/a	n/a	n/a	n/a	yes	CMHI1000, Sanger	*STXBP1*	c.[722C>A];[=]	p.[Ser241Tyr];[=]	de novo	AD	DEE4	DEVELOP
P31	F			n/a	n/a	1 m	n/a	n/a	n/a	n/a	n/a	n/a	n/a	CMHI1000	*AMT*	c.[452_466del];[452_466del]	p.[Lys151_Leu155del];[Lys151_Leu155del]	n/a	AR	GCE	METAB
P32	F			GE	n/a	n/a	n/a	no	no	n/a	n/a	no	yes	CMHI1000	*PCDH19*	c.[1145del];[=]	p.[Gly382Aspfs*187];[=]	n/a	XL	DEE9	DEVELOP
P33	F			GE	FEBR, TON-CLON	8 m	n/a	n/a	n/a	n/a	n/a	n/a	n/a	CMHI1000, Sanger	*SCN1A*	c.[2468A>T];[=]	p.[Asp823Val];[=]	de novo	AD	DS/DEE6B/GEFSP2	CHANNNEL
P34	F			FE	ABSEN	6 y	n/a	yes	yes	n/a	yes	yes	n/a	CMHI1000, Sanger	*CLN6*	c.[373A>G];[(373A>G)]	p.[Ser125Gly];[Ser125Gly]	biparental	AR	CLN	METAB
P35	F	yes		n/a	n/a	n/a	n/a	yes	yes	n/a	yes	n/a	n/a	WES	*SCN8A*	c.[2895C>A];[=]	p.[Asn965Lys];[=]	de novo	AD	DEE13	CHANNNEL
P36	F	yes		n/a	n/a	n/a	n/a	yes	n/a	n/a	yes	n/a	n/a	TSO	*SMC1A*	c.[3061dup];[=]	p.[Ala1021Glyfs*24];[=]	de novo	XLD	DEE85	DEVELOP
P37	M			n/a	n/a	2 d	no	n/a	yes	yes	n/a	yes	n/a	CMHI1000	*KCNQ2*	c.[1678C>T];[=]	p.[Arg560Trp];[=]	de novo	AD	DEE7	CHANNNEL
P38	M			n/a	IS	6 d	n/a	yes	yes	n/a	n/a	no	yes	CMHI1000, Sanger	*STXBP1*	c.[1162C>T];[=]	p.[Arg388*];[=]	n/a	AD	DEE4	DEVELOP
P39	M			n/a	n/a	3 y	n/a	yes	n/a	n/a	n/a	n/a	n/a	CMHI1000, Sanger	*SYNGAP1*	c.[3471G>A];[=]	p.[Trp1157*];[=]	de novo	AD	MRD5	DEVELOP
P40	F			n/a	n/a	5 y	n/a	yes	no	n/a	n/a	n/a	yes	CMHI1000, Sanger	*SLC2A1*	c.[667C>T];[=]	p.[Arg223Trp];[=]	paternal	AD	GLUT1DS	METAB
P41	F		yes	n/a	FEBR	6 m	n/a	n/a	no	n/a	n/a	n/a	n/a	CMHI1000, Sanger	*SCN1A*	c.[5285G>A];[=]	p.[Gly1762Glu];[=]	de novo	AD	DS	CHANNNEL
P42	M			n/a	n/a	n/a	n/a	yes	n/a	n/a	n/a	n/a	n/a	CMHI1000	*ATP1A2*	c.[889G>A];[=]	p.[Ala297Thr];[=]	n/a	AD	FHM2/DEE98	DEVELOP
P43	F			GE, FE	TON	5 m	yes	yes	n/a	n/a	yes	n/a	n/a	TSO, Sanger	*PCDH19*	c.[1031C>G];[=]	p.[Pro344Arg];[=]	paternal	XL	DEE9	DEVELOP
P44	F			FE	CLON	11 m	n/a	n/a	n/a	no	yes	no	yes	CMHI1000	*PCDH19*	c.[1091dup];[=]	p.[Tyr366Leufs*10];[=]	n/a	XL	DEE9	DEVELOP
P45	M			n/a	MYOCLON, TON-CLON	15 y	n/a	n/a	n/a	n/a	n/a	n/a	n/a	CMHI1000	*NHLRC1*	c.[660dup];[(660dup)]	p.[Val221Cysfs*13];[(Val221Cysfs*13)]	n/a	AR	MELF	METAB
P46	F			n/a	n/a	n/a	n/a	n/a	n/a	n/a	n/a	n/a	n/a	CMHI1000, Sanger	*SLC2A1*	c.[236G>T];[=]	p.[Gly79Val];[=]	de novo	AD	GLUT1DS	METAB
P47	M			GE	TON, CLON	3 y	yes	n/a	n/a	yes	n/a	yes	n/a	CMHI1000, Sanger	*SMARCB1*	c.[919T>C];[=]	p.[Phe307Leu];[=]	de novo	AD	CSS3	DEVELOP
P48	M			GE, FE	CLON	10 d	n/a	no	no	n/a	n/a	no	yes	CMHI1000	*ALDH7A1*	c.[328C>T];[328C>T]	p.[Arg110*];[Arg110*]	n/a	AR	EPEO4	METAB
P49	M			FE	CLON, MULTIPLE	2 d	n/a	n/a	n/a	n/a	n/a	n/a	yes	CMHI1000	*KCNQ2*	c.[821C>T];[=]	p.[Thr274Met];[=]	n/a	AD	DEE7	CHANNNEL
P50	F			n/a	n/a	1 m	n/a	no	no	no	n/a	no	n/a	TSO, Sanger	*ALDH7A1*	c.[328C>T];[902A>T]	p.[Arg110*];[Asn301Ile]	biparental	AR	EPEO4	METAB
P51	M	yes		n/a	TON, IS	3 d	n/a	yes	yes	n/a	yes	n/a	yes	CMHI1000	*KCNQ2*	c.[740C>T];[=]	p.[Ser247Leu];[=]	n/a	AD	DEE7	CHANNNEL
P52	M			GE, FE	n/a	12 m	yes	n/a	no	yes	n/a	n/a	yes	WES, Sanger	*ANKRD11*	c.[4387_4390del];[=]	p.[Glu1463Asnfs*67];[=]	de novo	AD	KBGS	DEVELOP
P53	M			n/a	n/a	12 m	n/a	n/a	yes	yes	yes	yes	yes	WES, Sanger	*WDR45*	c.[235+1G>T];[0]	p.[?];[0]	de novo	XLD	NBIA5	METAB
P54	M			FE	CLON	n/a	n/a	yes	yes	yes	yes	yes	n/a	CMHI1000, Sanger	*ATP1A2*	c.[1127C>T];[=]	p.[Thr376Met];[=]	n/a	AD	DEE98	DEVELOP
P55	M	yes		GE	TON, TON-CLON	2 d	n/a	yes	yes	n/a	yes	n/a	yes	CMHI1000, Sanger	*SCN2A*	c.[775C>A];[=]	p.[Leu259Ile];[=]	de novo	AD	DEE11	CHANNNEL
P56	M			GE	IS, TON	2 y	yes	yes	yes	yes	yes	yes	yes	CMHI1000, WES, Sanger	*UBA5*	c.[796G>C];[1111G>A]	p.[Val266Leu];[Ala371Thr]	biparental	AR	DEE44	DEVELOP
P57	M	yes		FE	CLON, TON, MYOCLON	2 m	n/a	n/a	n/a	n/a	yes	n/a	yes	CMHI1000	*SCN1A*	c.[626T>C];[=]	p.[Leu209Pro];[=]	n/a	AD	DEE6B	CHANNNEL
P58	F			n/a	TON, CLON	15 d	n/a	n/a	n/a	n/a	n/a	no	yes	CMHI1000	*KCNQ2*	c.[1678C>T];[=]	p.[Arg560Trp];[=]	n/a	AD	DEE7	CHANNNEL
P59	M		yes	GE	FEBR, CLON, TON	2.5 y	n/a	n/a	n/a	yes	yes	no	n/a	CMHI1000	*PCDH19*	c.[2452C>T];[=]	p.[Gln818*];[=]	n/a	XL	DEE9	DEVELOP
P60	F			n/a	FEBR	2 y	yes	yes	yes	yes	n/a	n/a	n/a	CMHI1000	*ANKRD11*	c.[7216C>T];[=]	p.[Gln2406*];[=]	n/a	AD	KBGS	DEVELOP
P61	F			GE, FE	ABSEN, MYOCLON, TON-CLON	6 y	yes	yes	yes	n/a	n/a	n/a	n/a	CMHI1000	*ZEB2*	c.[851G>A];[=]	p.[Cys284Tyr];[=]	n/a	AD	MOWS	DEVELOP
P62	F		yes	n/a	TON-CLON, FEBR	2 y	yes	yes	yes	n/a	no	no	no	CMHI1000	*KMT2A*	c.[1154del];[=]	p.[Lys385Argfs*15];[=]	n/a	AD	WDSTS	DEVELOP
P63	F			GE	ABSEN, FEBR	10 m	n/a	no	no	n/a	no	no	n/a	CMHI1000	*GABRG2*	c.[670C>T];[=]	p.[Arg224*];[=]	n/a	AD	DEE74	RECEPTOR
P64	F			GE	n/a	6 m	n/a	n/a	n/a	n/a	n/a	n/a	n/a	CMHI1000, Sanger	*PRRT2*	c.[649dup];[=]	p.[Arg217Profs*8];[=]	n/a	AD	BFIS2	DEVELOP
P65	F	yes		n/a	n/a	1 m	n/a	n/a	n/a	n/a	n/a	n/a	n/a	CMHI1000	*KCNQ2*	c.[436T>C];[=]	p.[Trp146Arg];[=]	n/a	AD	DEE7	CHANNNEL
P66	F			n/a	n/a	n/a	n/a	yes	n/a	yes	n/a	n/a	n/a	CMHI1000, Sanger	*CHD2*	c.[693-3C>A];[=]	p.[?];[=]	de novo	AD	DEE94	DEVELOP
P67	M			GE	TON-CLON, ATON	8 y	n/a	yes	n/a	n/a	n/a	n/a	n/a	CMHI1000	*STXBP1*	c.[560C>T];[=]	p.[Pro187Leu];[=]	n/a	AD	DEE4	DEVELOP
P68	F			n/a	TON	1 d	n/a	n/a	n/a	n/a	n/a	n/a	n/a	CMHI1000	*KCNQ2*	c.[1955dup];[=]	p.[Thr653Aspfs*212];[=]	n/a	AD	DEE7	CHANNNEL
P69	F			n/a	n/a	2 y	n/a	n/a	n/a	n/a	n/a	yes	n/a	CMHI1000, WES, Sanger	*UPB1*	c.[917-1G>A];[917-1G>A]	p.[?];[?]	biparental	AR	UPB1D	METAB
P70	F			GE	n/a	12 y	n/a	no	n/a	n/a	n/a	no	n/a	WES	*DEPDC5*	c.[4674G>A];[=]	p.[Trp1558*];[=]	n/a	AD	FFEVF1	DEVELOP
P71	F			n/a	TON-CLON	3 m	n/a	no	no	n/a	n/a	no	n/a	CMHI1000	*PRRT2*	c.[649dup];[=]	p.[Arg217Profs*8];[=]	n/a	AD	BFIS2	DEVELOP
P72	F			n/a	CLON, TON-CLON	4 y	yes	yes	yes	n/a	n/a	yes	yes	WES	*MECP2*	c.[1115C>A];[=]	p.[Ser372*];[=]	n/a	XLD	RTT	DEVELOP
P73	F		yes	GE	TON-CLON	6 m	n/a	n/a	yes	yes	n/a	no	yes	CMHI1000	*SCN1A*	c.[5590del];[=]	p.[Cys1864Valfs*13];[=]	n/a	AD	DS	CHANNNEL
P74	M	yes		FE	CLON	2 m	n/a	n/a	n/a	n/a	n/a	no	yes	CMHI1000	*SCN2A*	c.[3956G>C];[=]	p.[Arg1319Pro];[=]	n/a	AD	DEE11	CHANNNEL
P75	M			n/a	n/a	15 m	yes	yes	yes	yes	n/a	yes	yes	CMHI1000, Sanger	*OPHN1*	c.[1026-1G>T];[0]	p.[?];[0]	maternal	XLR	MRXSBL	DEVELOP
P76	M	yes		n/a	n/a	n/a	yes	yes	yes	yes	n/a	n/a	n/a	TSO, WES	*UBA5*	c.[698T>A];[796G>C]	p.[Leu233His];[Val266Leu]	biparental	AR	DEE44	DEVELOP
P77	M			n/a	ABSEN, IS, TON, CLON	3 m	n/a	n/a	no	yes	yes	yes	yes	CMHI1000	*TSC1*	c.[2228_2229del];[=]	p.[Gln743Argfs*10];[=]	n/a	AD	TSC	DEVELOP
P78	M			GE	TON-CLON, CLON, TON, MYOCLON, NOC, IS	4.5 y	n/a	yes	yes	yes	yes	n/a	yes	CMHI1000	*CHD2*	c.[2804_2813del];[=]	p.[Leu935Trpfs*2];[=]	n/a	AD	DEE94	DEVELOP
P79	F			n/a	MYOCLON	3 y	n/a	yes	yes	yes	yes	n/a	yes	CMHI1000	*KCTD7*	c.[190A>G];[249del]	p.[Thr64Ala];[Arg84Glyfs*25]	n/a	AR	EPM3	DEVELOP
P80	M			GE	ABSEN, ATON, TON-CLON	5 y	n/a	yes	n/a	yes	yes	no	yes	WES	*NPC1*	c.[1274C>A(;)3019C>G]	p.[Ser425*(;)Pro1007Ala]	n/a	AR	NPC	METAB
P81	M	yes		GE	MYOCLON	3 m	n/a	n/a	n/a	n/a	n/a	yes	n/a	CMHI1000	*KCNJ10*	c.[179T>C];[179T>C]	p.[Ile60Thr];[Ile60Thr]	n/a	AR	SESAMES	CHANNNEL
P82	F			n/a	n/a	n/a	n/a	yes	yes	yes	n/a	n/a	n/a	CMHI1000	*CUL3*	c.[655-1G>A];[=]	p.[?];[=]	n/a	AD	NEDAUS	DEVELOP
P83	F	yes		n/a	n/a	2 d	n/a	yes	yes	n/a	n/a	n/a	n/a	CMHI1000, Sanger	*PURA*	c.[885delinsGC];[=]	p.[His296Profs*21];[=]	de novo	AD	NEDRIHF	DEVELOP
P84	M	yes		n/a	MYOCLON, TON	3 d	n/a	n/a	n/a	n/a	n/a	n/a	yes	CMHI1000	*KCNQ2*	c.[619C>T];[=]	p.[Arg207Trp];[=]	n/a	AD	DEE7	DEVELOP
P85	M			FE	ABSEN	6 y	n/a	yes	yes	yes	n/a	yes	n/a	CMHI1000	*DYNC1H1*	c.[874C>T];[=]	p.[Arg292Trp];[=]	n/a	AD	CDCBM13	DEVELOP
P86	F			n/a	MYOCLON	12 m	n/a	n/a	n/a	yes	n/a	no	yes	CMHI1000	*CHD2*	c.[3499dup];[=]	p.[Val1167Glyfs*9];[=]	n/a	AD	DEE94	DEVELOP
P87	M			n/a	n/a	n/a	yes	yes	yes	yes	n/a	n/a	n/a	CMHI1000, Sanger	*ANKRD11*	c.[6191_6192del];[=]	p.(Ser2064Phefs*37)	de novo	AD	KBGS	DEVELOP

n/a, not available; F, Female; M, Male; CMHI1000, our Institute’s original NGS panel of >1000 genes related to pediatric diseases, TSO, TruSight One panel sequencing; WES, Whole Exome Sequencing; Sanger—Sanger sequencing; GE, generalized epilepsy; FE, focal epilepsy; MFE, multifocal epilepsy; TON, tonic seizures; CLON, clonic seizures; ATON, atonic seizures; MYOCLON, myoclonic seizures; IS, infantile spasm; ABSEN, absence seizures; TON-CLON, tonic–clonic seizures; MYOCLON-ATON, myoclonic–myoclonic–atonic seizures; MYOCLON-TON-CLON, myoclonic-tonic–clonic seizures; FEBR, febrile seizures/during infections; NOC, nocturnal seizures; MULTIPLE, multiple types of seizures, polymorphic seizures.

**Table 2:** Classification of epilepsies based on genetic/molecular pathomechanism.

	Classification of Epilepsies Based on Genetic/Molecular Pathomechanism			
	Channelopathies	Receptoropathies	Metabolic Epilepsy	Structural-Genetic/Developmental Epilepsy
Associated genes	*CACNA1A*, *KCNJ10*, *KCNQ2*, *SCN1A*, *SCN2A*, *SCN3A*, *SCN8A*	*GABRG2*, *GRIN2A*	*ALDH7A1*, *AMT*, *CLN6*, *GAMT*, *NHLRC1*, *NPC1*, *SLC2A1*, *TPP1*, *UPB1*, *WDR45*	*ALG13*, *ANKRD11*, *ATP1A2*, *ATP1A3*, *CDKL5*, *CHD2*, *COL4A1*, *CUL3*, *DEPDC5*, *DYNC1H1*, *ITPA*, *KANSL1*, *KCTD7*, *KMT2A*, *MECP2*, *OPHN1*, *PCDH19*, *POMT2*, *PRRT2*, *PURA*, *SMARCB1*, *SMC1A*, *SPTAN1*, *STXBP1*, *SYNGAP1*, *TSC1*, *TUBA1A*, *UBA5*, *ZEB2*
Number of genes (%)	7 (14.6%)	2 (4.2%)	10 (20.8)	29 (60.4%)
Number of patients (%)	26 (29.9%)	2 (2.3%)	13 (14.9%)	46 (52.9%)

## Data Availability

The original contributions presented in this study are included in the article/Supplementary Material. Further inquiries can be directed to the corresponding author.

## Supplementary Materials

The following supporting information can be downloaded at: https://www.mdpi.com/article/10.3390/genes17020133/s1.
